# Restoration of postictal cortical activity after electroconvulsive therapy relates to recovery of orientation in person, place, and time

**DOI:** 10.1192/j.eurpsy.2024.10

**Published:** 2024-02-14

**Authors:** Sven Stuiver, Julia C.M. Pottkämper, Joey P.A.J. Verdijk, Freek ten Doesschate, Michel J.A.M. van Putten, Jeannette Hofmeijer, Jeroen A. van Waarde

**Affiliations:** 1 Technical Medical Centre, Faculty of Science and Technology, Clinical Neurophysiology, University of Twente, Enschede, The Netherlands; 2 Department of Psychiatry, Rijnstate Hospital, Arnhem, The Netherlands; 3 Department of Neurology and Clinical Neurophysiology, Rijnstate Hospital, Arnhem, The Netherlands

**Keywords:** electroconvulsive therapy, electroencephalogram, recovery of orientation, consciousness, major depression

## Abstract

**Background:**

Most patients show temporary impairments in clinical orientation after electroconvulsive therapy (ECT)-induced seizures. It is unclear how postictal reorientation relates to electroencephalography (EEG) restoration. This relationship may provide additional measures to quantify postictal recovery and shed light on neurophysiological aspects of reorientation after ECT.

**Methods:**

We analyzed prospectively collected clinical and continuous ictal and postictal EEG data from ECT patients. Postictal EEG restoration up to 1 h was estimated by the evolution of the normalized alpha–delta ratio (ADR). Times to reorientation in the cognitive domains of person, place, and time were assessed postictally. In each cognitive domain, a linear mixed model was fitted to investigate the relationships between time to reorientation and postictal EEG restoration.

**Results:**

In total, 272 pairs of ictal-postictal EEG and reorientation times of 32 patients were included. In all domains, longer time to reorientation was associated with slower postictal EEG recovery. Longer seizure duration and postictal administration of midazolam were related to longer time to reorientation in all domains. At 1-hour post-seizure, most patients were clinically reoriented, while their EEG had only partly restored.

**Conclusions:**

We show a relationship between postictal EEG restoration and clinical reorientation after ECT-induced seizures. EEG was more sensitive than reorientation time in all domains to detect postictal recovery beyond 1-hour post-seizure. Our findings indicate that clinical reorientation probably depends on gradual cortical synaptic recovery, with longer seizure duration leading to longer postsynaptic suppression after ECT seizures.

## Introduction

Electroconvulsive therapy (ECT) has been used for the effective treatment of major depression since decades. In ECT sessions, a short electrical stimulus (<8 seconds, block pulse) is administered through (parts of) the patients’ brains to elicit self-terminating generalized seizure activity. The postictal state follows directly after seizure termination. During this state, patients may show temporary postictal symptoms, such as confusion with disorientation in person, place, or time [1–6]. Understanding the postictal state is important, because its duration has been related to both effectiveness and cognitive side effects of the ECT course [7–9].

During the postictal state, patients gradually regain orientation, typically from personal to spatial and temporal orientation [10]. At the bedside, recovery in these cognitive domains is clearly observed and classically assessed by the Reorientation Time (ROT) questionnaire [7]. Increased ROT values indicate a longer postictal state after an ECT session. This is clinically relevant, since longer ROT has been associated with poorer retrograde autobiographical memory outcomes, lasting from a week up to 6 months after the ECT course [7, 11]. On the other hand, longer ROT was associated with more rapid decline of depressive symptoms and better antidepressant outcomes of the ECT course [9].

The electroencephalogram (EEG) can be used to measure real-time cortical brain activity and also provides measures to quantify the postictal state. Immediately after termination of the ECT seizure, the postictal EEG shows suppression and slowing [6, 12, 13]. Postictal suppression is defined as amplitude reduction to less than 10 μV within 30 seconds of seizure transmission, lasting >2 seconds [13, 14]. Abnormal EEG slowing is indicated by an increase in the delta (1–4 Hz) and theta (4–7 Hz) frequency ranges and a reduction in faster frequencies [15, 16]. EEG recovery is probably more objective and more sensitive than ROT to quantify postictal recovery. Indeed, while clinical reorientation usually recovers within an hour, postictal EEG slowing may last up to 24 hours and it even takes longer before the EEG has completely returned to baseline activity [17, 18].

Currently, it is unknown how the restoration of the postictal EEG relates to recovery of clinical orientation after ECT. Such a link between EEG and clinical recovery may provide new objective and sensitive measures to quantify the postictal state. In addition, the relationship between clinical recovery and electrophysiological recovery may yield novel insights into the mechanisms of recovery of consciousness after seizures. Therefore, we studied whether postictal EEG restoration was related to recovery of clinical orientation by simultaneous continuous EEG recording and intermittent assessment of orientation in person, place, and time, immediately after ECT-induced seizures.

## Methods

### Study population

This is a *post hoc* exploratory analysis of prospectively collected trial data from 33 depressed patients treated with ECT in Rijnstate Hospital (Arnhem, The Netherlands), who participated in the StudY of effect of Nimodipine and Acetaminophen on Postictal Symptoms after ECT (SYNAPSE; NCT04028596). SYNAPSE was a randomized controlled trial with a three-condition crossover design. Patients received nimodipine, acetaminophen, or placebo (i.e., water) in a random and counterbalanced order at a maximum of 2 hours before each ECT session [19]. Repeated EEG measures before, during, and until 1 hour after the ECT sessions were collected to study the effects of the various treatment conditions on postictal recovery. Inclusion criteria for patients were age ≥18 years and having a current clinical diagnosis of major depression (i.e., classified as unipolar, bipolar, and schizoaffective disorder, according to the Diagnostic Manual of Mental Disorders, fifth edition [DSM-5]) [20]. The local medical ethical committee approved the study protocol (NCT04028596), and all included patients provided oral and written informed consent.

### ECT and treatment outcome measures

ECT was administered according to the Dutch treatment guidelines in the context of current care, twice a week, and electrodes were either placed right unilateral (RUL) or bi(fronto)temporal (BL) [21]. ECT stimuli were delivered with a constant current (900 mA), square wave, bidirectional, and brief pulse (1 millisecond) using the Thymatron System IV device (Somatics Incorporation, Lake Bluff, Illinois, USA). In case patients showed a poor response initially, RUL placement was switched to BL placement. Anesthesia was mostly achieved using etomidate (0.2–0.3 mg/kg body weight) for sedation and succinylcholine (0.5–1 mg/kg) for muscle relaxation. In case of severe postictal confusion, defined as the clinical necessity to administer sedatives or restraints due to severe motor restlessness, disorientation, agitation, or anxiety [22]), midazolam (2.5–5 mg intravenously) was used. Cessation of the ECT course was clinically decided by the treating psychiatrists and rated with the Hamilton Depression Rating Scale (HDRS) [23]. Clinical response was defined as a decrease of ≥50% of post-ECT HDRS score compared to pre-ECT, and remission was defined as a post-ECT HDRS score <8.

### EEG registration and preprocessing

Twelve or 20 silver/silver chloride cup electrodes were placed on the scalp according to the International 10–20 system. Cz was used as a reference. The electrode-skin impedance was kept below 5 kΩ. EEG was recorded before, during, and after the seizure: recordings started around 5 minutes before the administration of anesthesia and lasted up to 1 hour after seizure termination. In this study, both ictal and postictal EEG recordings were used. Data were band-pass filtered (0.5–30 Hz; first-order Butterworth filter) and segmented into 5-second epochs without artifacts. EEG recordings were visually inspected for artifacts by NeuroCenter EEG software (Leiden, The Netherlands). Electrodes containing noise or electrodes that were physically removed during the measurements (e.g., due to movement or postictal agitation) were rejected for analyses. All EEG analyses, preprocessing steps, and fits were conducted with MATLAB R2023a (MathWorks, Natick, MA, USA).

### Restoration of the postictal EEG

The start of the postictal state was defined at seizure termination, which was based on the cessation of spike-wave complexes and EEG suppression. Restoration of the postictal EEG was quantified with the normalized alpha–delta ratio (ADR), which was calculated and expressed as follows:(1)

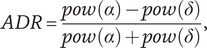

with 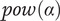 and 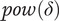 the power in the alpha (8–13 Hz) and delta (0.5–4 Hz) frequency bands, respectively. These values were calculated from computing power spectral density (PSD) values using Welch’s method [24]. Five-second artifact-free segments with an overlap of 50% were used, and median PSD values were determined for the available electrode channels (whole brain). ADR values were averaged per minute and fitted with a sigmoidal function given by(2)

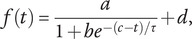

with 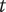 the duration of the postictal state (with a minimum duration of 40 min), 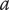 the distance from 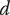 to the lower asymptote, 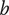 the transition from rapid to reduced growth, 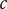 the initial lag, 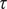 the time constant, and 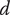 the upper asymptote. The values of the parameters ( 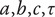 , and 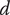 ) were estimated using a nonlinear least-squares routine. R^2^ ≥ 0.7 was considered appropriate for goodness of the fit. Subsequently, the derivative (df/dt) of equation 2 was calculated, given by(3)

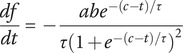

to estimate the timepoint where the recovery of ADR was at its maximum (T_max_). T_max_ was defined as the timepoint where equation 3 had its maximum value. The EEG features we used for postictal EEG restoration were the parameters 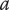 and 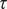 from equation 2 and T_max_ from equation 3.

### Seizure duration

Seizure duration was defined as the time interval between seizure onset and offset points, which were determined visually in the EEG. Seizure onset was defined as the onset of rhythmicity of spike-wave complexes, including waveform repetition with relatively uniform morphology and duration. Seizure offset was the timepoint where the seizure terminated, which was defined as the onset of generalized postictal suppression of at least 2 seconds [17, 25, 26].

### Recovery of clinical orientation

Before each ECT session, ROT questions were asked to assess orientation at baseline. After the ECT stimulus, ROT questions were asked every 5 minutes to assess recovery of clinical orientation [7]. The ROT questionnaire consists of five questions to assess orientation in person (name, birthday), place (name of hospital), and time (age, day of week). Time to reorientation in all three domains was determined separately by registration of the time (in minutes) until the question regarding a specific cognitive domain was answered correctly for the first time. Since the domains person and time consisted of two questions, these values were averaged, resulting in a single time to reorientation value for each domain. In case patients were not orientated at baseline in a specific domain, this domain was ignored for analysis.

### Statistical analyses

Variables were described by using numbers and percentages (%), means and standard deviation, and medians and interquartile range (IQR), as appropriate. Pre- and post-ECT HDRS scores were compared using Wilcoxon’s signed-rank test, with *p* < 0.05 regarded as statistically significant. Statistical analyses were computed using MATLAB and R version 4.3.1 using the lme4 and report packages [27, 28].

#### Recovery of clinical orientation

Time to reorientation values in the three cognitive domains person, place, and time were averaged (median and IQR) across subjects. Wilcoxon’s signed-rank tests were used to test whether these values in person, place, and time differed.

#### Restoration of the postictal EEG and recovery of clinical orientation

Because of the inherently correlated data structure, three linear mixed models (LMMs) were fitted to predict clinical postictal recovery, one for each. Fixed effects, derived from the postictal EEG, were T_max_, 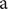 , and 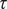 . Fixed effects related to patient and ECT parameters were electrical charge of the ECT stimulus (in millicoulombs [mC]), seizure duration (in seconds), administration of midazolam, the applied electrode placement, and the number of ECT sessions of the ROT measurement, sex, and age (in years). Random effects (slope and intercept) were subject and T_max_, in which specification was chosen based on avoiding singularity [29]. Separate analyses were performed in measurements without midazolam to account for its known effects on EEG recordings [30].

#### Restoration of the postictal EEG and ECT parameters

For each postictal EEG feature (i.e., T_max_, 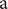 , and 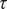 ), LMMs were fitted to predict postictal restoration, one for each (Supplementary Table S3).

LMMs assume that random effects deviations and residual errors are normally distributed, which were verified. All fixed effects were checked for multicollinearity, where a variance inflation factor <5 was considered as a low correlation and tolerable. Because of the relatively small sample size (N = 33), the restricted maximum likelihood was used as an estimation method. 95% confidence interval (CI) and *p*-values (alpha = 0.05) were computed using a Wald t-distribution approximation.

## Results

### Patient and treatment characteristics

Out of the SYNAPSE database (N = 33), we included 272 pairs of ictal-postictal EEGs and clinical measures from 32 patients, because one patient had to be excluded due to poor EEG quality. Patient and treatment characteristics are shown in Table 1. The mean age was 54.2 ± 13.9 years, and 18 patients (56%) were female. Postictal EEG recordings of 90 ECT sessions (34.2%) were registered after RUL stimulation and the remaining after BL stimulation. Immediately after 101 ECT sessions (37.1%; N = 13 patients), midazolam was administered intravenously. At the group level, patients received a median of 14 (IQR = 4.9) ECT sessions during the completed ECT course, with a mean applied electrical charge of 349.3 ± 162.4 mC. The mean seizure duration was 58.5 ± 29.9 seconds. HDRS scores decreased significantly after the ECT course (median pre-ECT HDRS score = 23.5, IQR = 10; median post-ECT HDRS score = 12, IQR = 9; *p* < 0.001). After the ECT course, 16 patients (50%) showed clinical response and seven (22%) remission.

**Table 1.** Clinical and demographic characteristics of patients (*N* = 32)

Age, mean ± SD	54.2 ± 13.9
Sex, female (%)	18 (56)
Pre-ECT HDRS score, median (IQR)	23.5 (10)^a^
Electrode placement, RUL (%)	90 (34.2)
Electrical charge, mean ± SD, in millicoulombs	349.3 ± 162.4
Post-ECT HDRS score, median (IQR)	12 (9)^a^
Clinical response: decrease of ≥50% of post-ECT HDRS score compared to pre-ECT, *n* (%)	16 (50)
Clinical remission: post-ECT HDRS score <8, *n* (%)	7 (22)
Total number of ECT sessions in the course, median (IQR)	14 (4.9)

Abbreviations: ECT, electroconvulsive therapy; HDRS, Hamilton Depression Rating Scale; IQR, interquartile range; RUL, right unilateral; SD, standard deviation.

a Wilcoxon’s signed-rank test, *p* < 0.001.

### Restoration of postictal EEG

**Figure 1.**
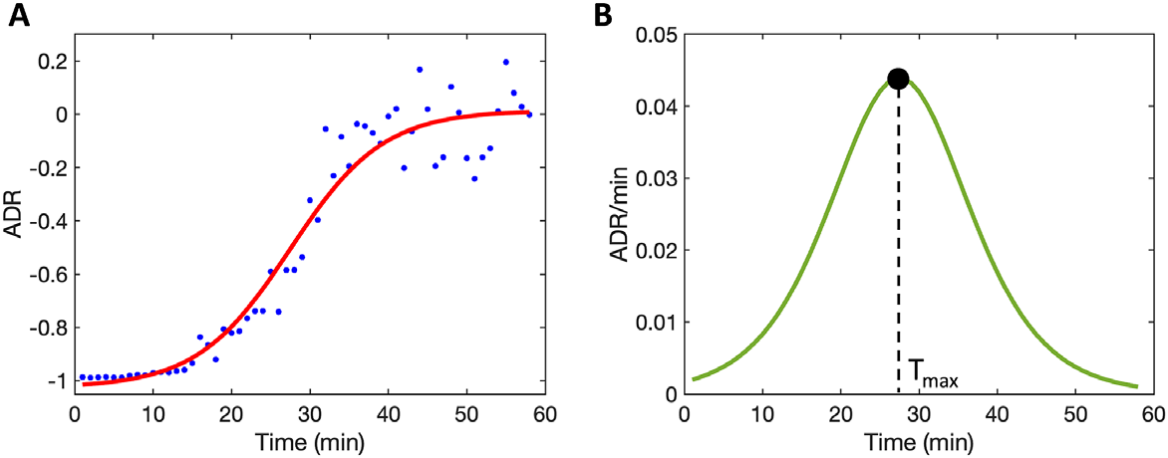
Restoration of the postictal electroencephalogram (EEG) expressed as the evolution of the normalized alpha–delta ratio (ADR; equation 1). (A) Averaged ADR values per minute (blue dots) followed a typical sigmoidal evolution in the postictal state, starting at values close to −1. The evolution of ADR is well described by the sigmoidal curve (solid red line), defined by equation 2. Here, 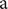 = 1.0 ± 0.1 ( 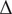 ADR) and 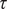 = 5.9 ± 4.4 min. (B) From the derivative (green solid line), defined by equation 3, T_max_ is estimated. Here, T_max_ = 27 min. These data are from subject 2 in this study.

The typical recovery of the postictal EEG in the first hour followed a sigmoid-like recovery of ADR (Figure 1). At the group level, averaged values for postictal EEG parameters were 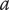 = 0.33 ± 0.27 ( 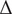 ADR), 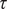 = 6.3 ± 3.1 min, and T_max_ = 29.4 ± 8.6 min. While ADR values increased over time during the postictal state, no patient reached baseline ADR values within 1 hour after the seizure.

### Recovery of clinical orientation

**Figure 2.**
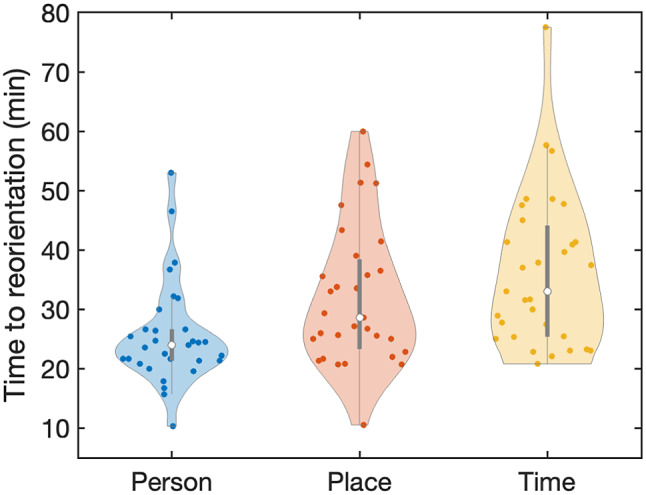
Median time to reorientation values in person, place, and time after electroconvulsive therapy (ECT)-induced seizures. Patients firstly recovered in person (median = 24.0 min, IQR = 5.2) compared to the domains place (median = 28.6 min, IQR = 15.0 min, *p* < 0.001) and time (median = 33.0 min, IQR = 18.7, *p* < 0.001). Reorientation in place was faster than reorientation in time (*p* = 0.012).

In Figure 2, violin plots of the median time to reorientation values in person, place, and time are shown. At the group level, patients recovered firstly in person (median = 24.0 min, IQR = 5.2, *p* < 0.001), compared to time to reorientation in place (median = 28.6 min, IQR = 15.0, *p* < 0.001) and time to reorientation in time (median = 33.0 min, IQR = 18.7, *p* < 0.001). Also, patients recovered faster in place compared to reorientation in time (*p* = 0.012). Nineteen patients (61%) reached recovery of orientation in the exemplary sequence: person-place-time. None of the patients showed reorientation in time before reorientation in the other two domains.

### Restoration of postictal EEG in relation to recovery of clinical orientation

#### Timepoint of maximum recovery (Tmax)

**Figure 3.**
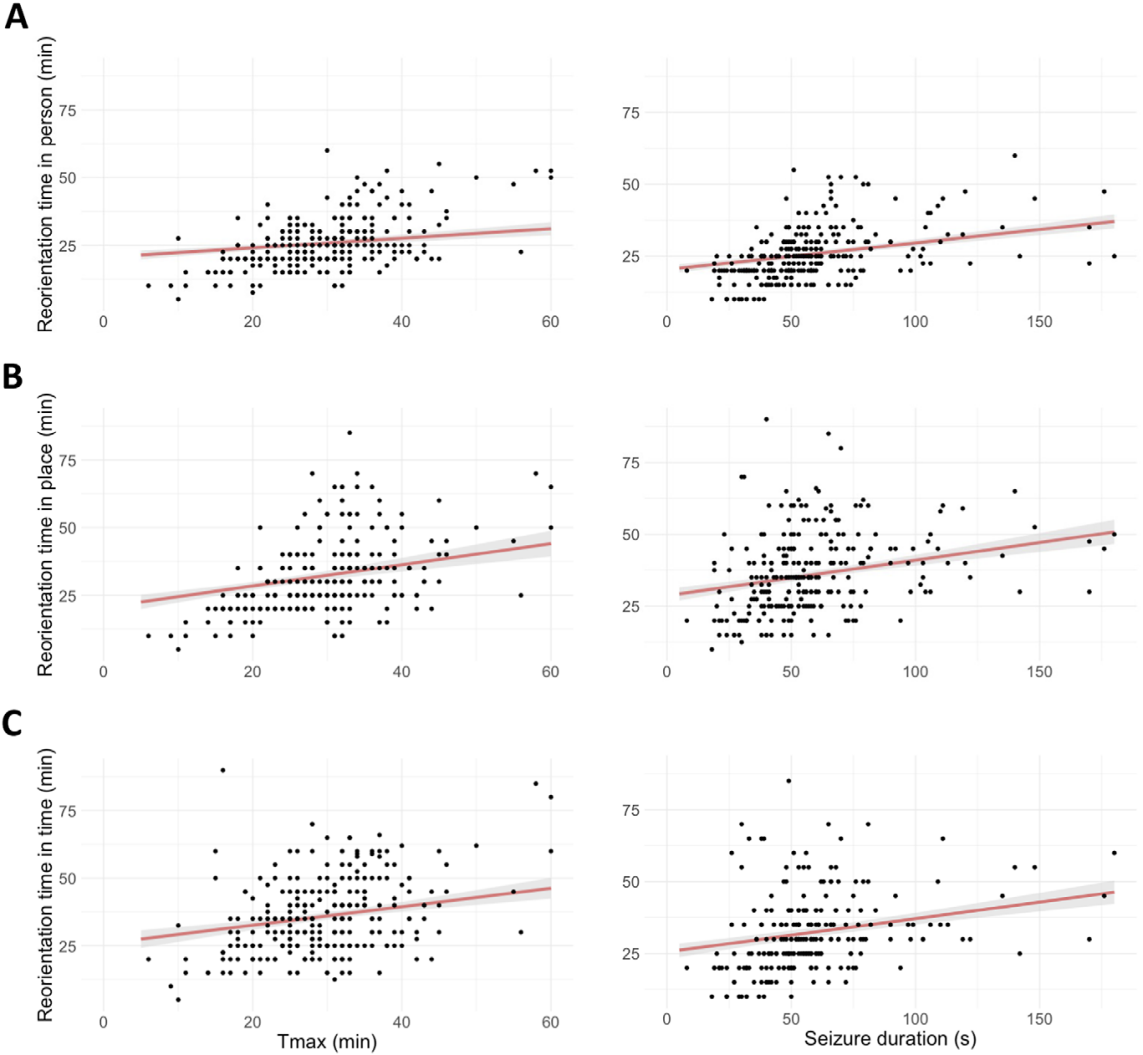
Positive correlations between T_max_ (i.e., the timepoint where the recovery of the electroencephalogram [EEG] was maximized) and seizure duration and time to reorientation in three cognitive domains (i.e., person [A], place [B], and time [C]). Marginal effects (red lines) and 95% CI (shaded errors) are estimated from the models.

In all three cognitive domains, relationships between T_max_ and time to reorientation were observed (see Figure 3), i.e., positive relations between T_max_ and time to reorientation in person (β = 1.5, 95% CI [0.4, 2.6], *p* = 0.007), place (β = 3.3, 95% CI [1.3, 5.4], *p* = 0.002), and time (β = 2.4, 95% CI [0.5, 4.4], *p* = 0.016).

#### *Extent of recovery (* 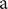 *)*

Negative relations were found between the extent of recovery ( 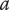 ) and time to reorientation in person (β = −1.2, 95% CI [−2.3, −0.1], *p* = 0.036) and in time (β = −2.8, 95% CI [−4.8, −0.8], *p* = 0.007). No relationship was found with reorientation in place (β = −1.2, 95% CI [−3.0, 0.7], *p* = 0.171).

#### *Time constant (* 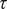 *)*

No relationships were found between time constant 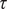 and time to reorientation for all three domains (in person: β = −0.7, 95% CI [−0.1, 1.5], *p* = 0.094; in place: β = −0.7, 95% CI [−2.0, 0.6], *p* = 0.300; and in time: β = −0.3, 95% CI [−1.8, 1.2], *p* = 0.699).

### Seizure duration in relation to recovery of clinical orientation

Positive relations were found between seizure duration and time to reorientation (in person β = 2.5, 95% CI [1.5; 3.5], *p* < 0.001); in place (β = 3.1, 95% CI [1.5, 4.8], *p* < 0.001); and in time (β = 3.3, 95% CI [1.6, 5.1], *p* < 0.001) (Figure 3).

### Recovery of orientation in relation to patient and ECT parameters

#### Age

Higher age was related to longer duration of reorientation in time (β = 4.0, 95% CI [1.3, 6.7], *p* = 0.004). No significant associations were found in the other two domains.

#### Electrode placement

BL (compared to RUL) electrode placement seemed associated with longer time to reorientation in place (β = 5.2, 95% CI [−0.4, 10.8], *p* = 0.070); however, this finding was not significant. In the other two domains, no associations were found.

#### Electrical charge of the ECT stimulus

The electrical charge of the ECT stimulus was positively related to time to reorientation in place (β = 2.6, 95% CI [0.5, 4.8], *p* = 0.014). No significant associations were found in the other two domains.

#### Postictal midazolam

Administration of midazolam was associated with longer time to reorientation in person (β = 4.4, 95% CI [1.3, 7.4], *p* = 0.005), in place (β = 8.3, 95% CI [3.5, 13.2], *p* < 0.001), and in time (β = 5.6, 95% CI [0.4, 10.7], *p* = 0.034).

The results of all fixed-effects and random-effects designs, formulas, and model performances of the LMMs for recovery of clinical orientation in all three cognitive domains (i.e., person, place, and time) are shown in Supplementary Table S1. Analyses of data from measurements without midazolam (N = 171 measurements; N = 20 subjects) showed similar results (Supplementary Table S2). In Supplementary Table S3, associations between the postictal EEG features (T_max_, 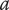 , 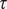 ) and other patient and ECT characteristics are shown.

## Discussion

In this analysis of prospectively collected clinical and EEG data, the gradual restoration of the postictal EEG was related to gradual recovery of clinical orientation in all three cognitive domains (i.e., in person, place, and time) after ECT-induced seizures. Slower restoration of postictal EEG was associated with longer time to reorientation in all domains. Longer seizure duration and postictal administration of midazolam were related to later time to reorientation. Clinical reorientation mostly recovered within 1 hour after the ECT stimulus, but ADR values never reached baseline values in that time frame. Our findings show that EEG is more sensitive than ROT to measure postictal recovery at 1 hour post-seizures and suggest that recovery of orientation after ECT is related to the gradual restoration of cortical synaptic recovery.

Derived from the postictal EEG, the parameters 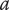 (indicating the extent of EEG restoration) and T_max_ (indicating the timepoint where EEG restoration was at its maximum) were related to ROT values. The EEG is dominated by postsynaptic potentials, and restoration of the postictal EEG probably reflects the gradual recovery of cortical synaptic activity. Alpha oscillations have been theorized to play a key role in cognition and are present during (relaxed) wakefulness [31]. Restoration of this rhythm, indicated by an increase in ADR, probably relates to recovery of cortical synaptic activity. Our chosen EEG feature, the ADR, serves as a proxy for this postictal EEG restoration, which as we show typically follows a sigmoidal function in the first hour. In most patients, clinical orientation recovered within an hour, while ADR values never normalized. This shows that a clinical psychometric instrument, such as the ROT [7], has ceiling effects and that EEG provides a more sensitive measure to estimate recovery of the postictal state. Previous work has shown that longer ROT is associated with retrograde amnesia, an important side effect of ECT [7, 11]. We hypothesize a similar relationship with slower restoration of the postictal EEG, which may be a more sensitive measure for predicting retrograde amnesia. Our observed relationships between postictal EEG features and patient and ECT parameters support this hypothesis. EEG recordings beyond 1 hour post-seizure may reveal important aspects (including prolonged disturbances of brain activity) of the postictal state that are currently neglected by clinical measures of reorientation.

Longer seizure duration was related to later time to reorientation in all domains. This finding is in line with previous findings [17]. During a seizure, there is an increase in energy consumption and cerebral blood flow, oxygen consumption, and glucose metabolism [32, 33]. Prolonged seizure activity may result in more (local) deprivation of oxygen and energy, inducing longer postictal synaptic depression and slower cortical synaptic recovery. Our results show a larger effect size of seizure duration on time to reorientation with an increase in complexity of the cognitive domain, from person to place to time. It has been shown that amnestic effects of ECT are greatest for impersonal memory (i.e., knowledge about the world) compared to personal memory, for recent compared to distinctly remote events, and for less salient events [34]. Therefore, domains involving impersonal memory and more recently stored information (i.e., orientation in time and place) may be more vulnerable to the effects of ECT-induced seizures, especially in longer seizures.

More than half of the patients recovered in orientation in the sequence person-place-time and never in time before the other two domains. The specific order of reorientation in person-place-time is in line with earlier findings [10, 35] and was also observed in patients recovering from closed head injury [36]. Furthermore, this finding is also in line with observations in patients with dementia, where orientation disappears in counterorder [37]. The sequence of recovery in person-place-time may again point toward differences between personal and recently stored information, or at distinct restoration processes of consciousness, possibly in different parts of the human brain or functional networks [37].

Previous research found that the length of postictal disorientation tends to increase with more numbers of previous ECT sessions and with the application of BL electrode placement [35]. In this study, we show that higher age was related to longer duration of reorientation in time. Furthermore, higher applied electrical charge and BL electrode placement (and at trend level with RUL, *p* = 0.070) were related to longer ROT in place. According to modeling studies, these ECT parameters determine the spread of current density in the brain during electrical stimulation [38]. Postictal administration of midazolam was associated with later time to reorientation in all domains. The use of this agent is known to increase delta power and decrease alpha power [30]. Therefore, midazolam may delay postictal ADR restoration, resulting in longer ROTs. Separate analyses without midazolam showed similar findings regarding the relationships between postictal EEG and clinical reorientation. Our findings indicate that some patient characteristics and ECT parameters may only affect ROT values of specific domains, while others show persistent effects in all domains of orientation.

### Strengths and limitations

Strengths of this study include the prospective collection of large number of repeated measures of ictal and postictal EEG data (N = 272), the use of continuous EEG monitoring, and the analysis of time to reorientation values in three domains. Furthermore, this study included systematically measured postictal brain activity up to 1 hour after seizure termination together with direct clinical measures of reorientation. However, some limitations apply to this study. First, the study was not powered for this post hoc evaluation of time to reorientation in the three distinct cognitive domains. Second, we did not account for possible confounding effects of the concomitant use of medication (i.e., antidepressants, antipsychotics, antiepileptics, and benzodiazepines) during the ECT course. Finally, the remission rate of ECT in this study cohort was low (22%), compared to reported ECT remission rates of 51–58% [39], probably due to the patient selection for ECT in the Netherlands (i.e., often only indicated as treatment of last resort).

## Conclusion

We found a clear relationship between clinical recovery of orientation and restoration of EEG activity after ECT-induced seizures. Longer time to reorientation was related to slower restoration of the postictal EEG. Despite complete clinical reorientation, the EEG showed enduring postictal disturbances at 1 hour post-seizures, pointing toward a higher sensitivity of EEG than ROT to estimate postictal recovery. In addition, longer seizure duration and postictal administration of midazolam were related to increased time to reorientation values. Our results imply that clinical reorientation probably depends on gradual cortical synaptic recovery and that longer seizures lead to longer postsynaptic suppression.

## Supporting information

Baenas et al. supplementary material Baenas et al. supplementary material

## Supplementary material

The supplementary material for this article can be found at https://doi.org/10.1192/j.eurpsy.2024.10.
