# Supplementary material for: Restoration of postictal cortical activity after electroconvulsive therapy relates to recovery of orientation in person, place, and time
Source: Eur Psychiatry. 2024 Feb 14;67(1):e16. doi: 10.1192/j.eurpsy.2024.10 (PMC10966617; doi:10.1192/j.eurpsy.2024.10)
Supplement: Baenas et al. supplementary material [file S0924933824000105sup001.docx]

**Supplementary material**

**Table S1.** Results of fixed effects of the linear mixed models (LMMs) for recovery of clinical orientation in the domains in person, place and time. Values represent means with 95% upper and lower confidence intervals. Postictal medication (i.e., midazolam), electrode placement and sex were binarized (with value 1 = use of postictal medication, 1 = bi(fronto)temporal stimulation, and 1 = male, respectively).

|  | **Intercept** | **T_max_** | $\boldsymbol{a}$ | $\boldsymbol{\tau}$ | **Electrical charge** | **Seizure duration** | **Postictal medication** | **Electrode placement** | **ECT-session** | **Sex** | **Age** |
| --- | --- | --- | --- | --- | --- | --- | --- | --- | --- | --- | --- |
| **ROT in person** | 24.2 [20.2, 28.3]*** | 1.5 [0.4, 2.6]** | -1.2 [-2.3, -0.1]* | 0.7 [-0.1, 1.5] | -0.3 [-1.7, 1.1] | 2.5 [1.5, 3.5]*** | 4.4 [1.3, 7.4]** | 1.3 [-2.3, 4.9] | 0.1 [-0.1, 0.3]* | -2.9 [-7.1, 1.3] | -0.3 [-2.2, 1.7] |
| **ROT in place** | 25.1 [18.9, 31.4]*** | 3.3 [1.3, 5.4]** | -1.2 [-3.0, 0.5] | -0.7 [-2.0, 0.6] | 2.6 [0.5, 4.8]* | 3.1 [1.5, 4.8]*** | 8.3 [3.5, 13.2]*** | 5.2 [-0.4, 10.8] | -0.1 [-0.4, 0.3] | 1.9 [-4.0, 7.9] | 0.2 [-2.5, 2.9] |
| **ROT in time** | 31.2 [25.2, 37.2]*** | 2.9 [0.9, 4.9]** | -2.8 [-4.8,-0.8]** | -0.3 [-1.8, 1.2] | 0.5 [-1.9, 3.0] | 3.3 [1.6, 5.1]*** | 5.6 [0.4, 10.7]* | 2.5 [-3.1, 8.2] | -0.2 [-0.6, 0.3] | 3.5 [-2.5, 9.4] | 4.0 [1.3, 6.7]** |

ROT = reorientation time; T_max_ = timepoint in the postictal electroencephalogram where recovery is maximized; $a$ = the extent of recovery ($\Delta$ADR [alpha/delta ratio]); $\tau$ = time constant; ECT = electroconvulsive therapy; *p < .05; **p < .01; ***p < .001

**Model design and performances**

*Model design.* A linear mixed model (LMM) to predict time to reorientation for each domain (i.e., person, place and time, in minutes) with fixed effects T_max_, $a$, $\tau$, electrical charge of the ECT-stimulus (in millicoulombs), seizure duration (in seconds), postictal medication (1 = use of postictal medication), electrode placement (1 = bi[fronto]temporal stimulation) and number of ECT-session, sex (1 = male) and age (in years) was fitted. T_max_ and subject were included as random effects. The formula was: ‘ROT ~ T_max_ + $a$ + $\tau$ + electrical charge + seizure duration + postictal medication + electrode placement + number of ECT-session + sex + age + (1 + T_max_ | subject)’.

*Model performance.* For time to reorientation in person, the model’s total explanatory power (i.e., conditional R^2^) was 0.72 and the part related to the fixed effects (i.e., marginal R^2^) alone was 0.35. For time to reorientation in place and time, conditional R^2^ was 0.75 and 0.55, and marginal R^2^ was 0.31 and 0.29, respectively.

**Table S2.** Results of fixed effects of the linear mixed models (LMMs) for recovery of clinical orientation in the domains in person, place and time without midazolam. Values represent means with 95% upper and lower confidence intervals. Electrode placement and sex were binarized (with value 1 = bi(fronto)temporal stimulation and 1 = male, respectively).

|  | **Intercept** | **T_max_** | $\boldsymbol{a}$ | $\boldsymbol{\tau}$ | **Electrical charge** | **Seizure duration** | **Electrode placement** | **ECT-session** | **Sex** | **Age** |
| --- | --- | --- | --- | --- | --- | --- | --- | --- | --- | --- |
| **ROT in person** | 26.9 [21.4, 30.6]*** | 1.4 [0.3, 2.5]* | -1.2 [-2.5, 0.1] | 0.4 [-0.5, 1.4] | 03 [-1.3, 1.9] | 3.1 [1.8, 4.4]*** | -1.6 [-5.6, 2.4] | 0.1 [-0.2, 0.4] | -4.0 [-8.6, 0.6] | -0.2 [-2.3, 1.9] |
| **ROT in place** | 26.6 [19.2, 34.0]*** | 4.3 [1.5, 7.0]** | -0.5 [-2.7, 1.7] | -0.8 [-2.5, 1.0] | 2.8 [0.3, 5.4]* | 3.8 [1.4, 6.3]** | 3.9 [-2.7, 10.5] | -0.2 [-0.7, 0.4] | 1.2 [-5.6, 7.9] | 0.3 [-2.9, 3.4] |
| **ROT in time** | 32.4 [24.5, 40.3]*** | 2.2 [-0.2, 4.6] | -2.9 [-5.6, -0.2]* | -0.8 [-2.9, 1.4] | 2.3 [-1.0, 5.5] | 4.6 [2.0, 7.1]*** | 3.3 [-3.6, 10.2] | -0.5 [-1.2, 0.1] | 2.8 [-4.6, 10.2] | 3.4 [0.0, 6.8]* |

ROT = reorientation time; T_max_ = timepoint in the postictal electroencephalogram where recovery is maximized; $a$ = the extent of recovery ($\Delta$ADR [alpha/delta ratio]); $\tau$ = time constant; ECT = electroconvulsive therapy; *p < .05; **p < .01; ***p < .001

*Model design.* Similar to Table S1 but without midazolam as fixed effect.

*Model performance.* For time to reorientation in person, the model’s total explanatory power (i.e., conditional R^2^) was 0.71 and the part related to the fixed effects (i.e., marginal R^2^) alone was 0.30. For time to reorientation in place and time, conditional R^2^ was 0.72 and 0.49, and marginal R^2^ was 0.21 and 0.25, respectively.

**Table S3.** Results of fixed effects of the linear mixed models (LMMs) for postictal EEG restoration parameters T_max_, $a$ and $\tau$. Values represent means with 95% upper and lower confidence intervals. Postictal medication (i.e., midazolam), electrode placement and sex were binarized (with value 1 = use of postictal medication, 1 = bi(fronto)temporal stimulation, and 1 = male, respectively).

|  | **Intercept** | **Electrical charge** | **Seizure duration** | **Postictal medication** | **Electrode placement** | **ECT-session** | **Sex** | **Age** |
| --- | --- | --- | --- | --- | --- | --- | --- | --- |
| **T_max_** | 26.8 [23.4, 30.3]*** | 1.0 [-0.5, 2.4] | 3.9 [2.4, 5.4]*** | 5.5 [2.6, 8.4]*** | -1.5 [-4.7, 1.8] | 0.3 [0.0, 0.6]* | 0.4 [-2.9, 3.7] | -0.6 [-2.1, 0.9] |
| $\boldsymbol{a}$ | 0.60 [0.47, 0.73]*** | -0.07 [-0.11, -0.02]** | -0.04 [-0.07, -0.01]* | -0.04 [-0.14, 0.06] | -0.15 [-0.27, -0.03]* | -0.02 [-0.02, -0.01]*** | -0.07 [-0.22, 0.08] | 0.06 [-0.01, 0.12] |
| $\boldsymbol{\tau}$ | 6.4 [5.1, 7.8]*** | -0.3 [-0.9, 0.3] | 0.1 [-0.3, 0.3] | -0.5 [-1.7, 0.7] | -0.5 [-1.8, 0.8] | 0.2 [0.0, 0.3]* | -1.0 [-2.3, 0.4] | -0.2 [-0.8, 0.4] |

T_max_ = timepoint in the postictal electroencephalogram where recovery is maximized; $a$ = the extent of recovery ($\Delta$ADR [alpha/delta ratio]); $\tau$ = time constant; ECT = electroconvulsive therapy; *p < .05; **p < .01; ***p < .001

Seizure duration (β = 3.90, 95% CI [2.4, 5.4], *p* < .001) and number of the ECT-session (β = 0.29, 95% CI [0.0, 0.6], *p* = .036) were positively related with T_max_. Administration of midazolam was positively associated with T_max_ (β = 5.5, 95% CI [2.6, 8.4], *p* < .001). Electrical charge of the ECT-stimulus (β = -0.07, 95% CI [-0.11, -0.02], *p* = .004) and seizure duration (β = -0.04, 95% CI [-0.07, -0.01], *p* = .020) were negatively related to $a$. BL electrode placement (β = -0.15, 95% CI [-0.27, -0.03], *p* = .013) and ECT-session number (β = -0.02, 95% CI [-0.02, -0.01], *p* < .001) were negatively associated with $a$. ECT-session number was positively associated with $\tau$ (β = 0.2, 95% CI [0.0, 0.3], *p* = .015). Results of fixed effects and model performances are shown in Supplementary Table S3.

**Model design and performances**

*Model design.* A linear mixed model (LMM) to predict postictal EEG features T_max_, $a$, $\tau$ (one for each) with fixed effects electrical charge of the ECT-stimulus (in millicoulombs), seizure duration (in seconds), postictal medication (1 = use of postictal medication), electrode placement (1 = bi[fronto]temporal stimulation) and number of ECT-session, sex (1 = male) and age (in years) was fitted. Seizure duration and subject were included as random effects. The formula was: ‘T_max_/$a$/$\tau$ ~ electrical charge + seizure duration + postictal medication + electrode placement + number of ECT-session + sex + age + (1 + seizure duration| subject)’.

*Model performance.* For T_max_, the model’s total explanatory power (i.e., conditional R^2^) was 0.52 and the part related to the fixed effects (i.e., marginal R^2^) alone was 0.33. For $a$ and $\tau$, conditional R^2^ was 0.65 and 0.18, and marginal R^2^ was 0.18 and 0.04, respectively.
